# Diagnosing Musculoskeletal Tumours

**DOI:** 10.1155/S1357714X01000172

**Published:** 2001-06

**Authors:** Robert J. Grimer, Simon R. Carter, David Spooner, Rodney S. Sneath

**Affiliations:** 1 The Royal Orthopaedic Hospital Oncology Service Birmingham UK; 2 The Royal Orthopaedic Hospital Oncology Service The Royal Orthopaedic Hospital Bristol Road South, Northfield Birmingham B31 2AP UK

## Abstract

In 1993 we became aware of a worrying increase in apparent errors in the histopathological diagnosis of musculoskeletal tumours in our Unit. As a result all cases seen over the past 8 years were reviewed by an independent panel. Of the 1996 cases reviewed there was an error in 87. In 54 cases (2.7%) this had led to some significant change in the active management of the patient. The main areas where errors arose were in those very cases where clinical and radiological features were not helpful in confirming or refuting the diagnosis. The incidence of errors rose with the passage of time, possibly related to a deterioration in the pathologist’s health. The error rate in diagnosing bone tumours in previously published series ranges from 9 to 40%. To ensure as accurate a rate of diagnosis as possible multidisciplinary working and regular audit are essential.


*Sarcoma* (2001) 5, 89–94

ORIGINAL ARTICLE

### Diagnosing musculoskeletal tumours

ROBERT J. GRIMER, SIMON R. CARTER, DAVID SPOONER & RODNEY S. SNEATH

*The Royal Orthopaedic Hospital Oncology Service, Birmingham, UK*

**Abstract**

In 1993 we became aware of a worrying increase in apparent errors in the histopathological diagnosis of musculoskeletal tumours in our Unit. As a result all cases seen over the past 8 years were reviewed by an independent panel. Of the 1996 cases reviewed there was an error in 87. In 54 cases (2.7%) this had led to some significant change in the active management of the patient. The main areas where errors arose were in those very cases where clinical and radiological features were not helpful in confirming or refuting the diagnosis. The incidence of errors rose with the passage of time, possibly related to a deterioration in the pathologist's health. The error rate in diagnosing bone tumours in previously published series ranges from 9 to 40%. To ensure as accurate a rate of diagnosis as possible multidisciplinary working and regular audit are essential.

### Introduction

There is little doubt that diagnosing musculoskeletal tumours is far from straightforward. Huvos states in the introduction to his book that 'diagnosis and treatment of bone tumours is as much an art as a science',[1] whilst Schajowicz pointed out that 'some lesions still present serious and in part unresolved problems of diagnosis even to the experienced pathologist'.[2]

In recent years there have been a number of articles and editorials highlighting not only the difficulties of biopsying musculoskeletal tumours but also commenting on the error rates in pathological diagnosis of these tumours.[3–8]

The overwhelming message from these papers is the belief that it is necessary for the biopsy to be carried out in the centre where definitive treatment is going to be provided. The principle reasons for this belief are that:

1. appropriate pre biopsy staging can be completed;
2. the optimum method and site of biopsy can be chosen;
3. the biopsy does not compromise subsequent definitive surgical options;
4. there is the lowest rate of complications following the biopsy;
5. a pathologist experienced in diagnosing musculoskeletal tumours can interpret the biopsy.

These guidelines have been available for many years yet biopsies are still performed suboptimally. Mankin *et al.* in their recent review of biopsy problems[7] found that there had been no significant improvement in referral patterns or accuracy of diagnosis over a 10-year period.

The actual accuracy of the histopathological diagnosis has also been commented on in most of the papers mentioned and error rates have varied from 9 to 36%. All of these error rates have either been based upon identifiable errors which have subsequently become apparent or on alterations in diagnosis at national or local tumour registries.

We report here the results of an independent audit of one Unit's work to show the errors that have occurred over an 8-year period.

### Background

In 1993 it became apparent that there was a worrying level of possible inaccuracies in histopathological reports relating to musculoskeletal tumours at our Unit. It was decided that an independent enquiry should be instituted and following the recommendations of this enquiry a review of all cases seen and treated over the previous 8 years was carried out. Recognised experts in musculoskeletal histopathology were invited to review the cases and this process took almost 2 years. In any case where an error in diagnosis was identified that case was double checked by another pathologist. An independent clinical advisory group consisting of an experienced pathologist, radiologist, surgeon and oncologist also advised whether there was any detriment to the

Correspondence to: Mr R.J. Grimer FRCS, The Royal Orthopaedic Hospital Oncology Service, The Royal Orthopaedic Hospital, Bristol Road South, Northfield, Birmingham B31 2AP, UK. Tel.: +44-121-685-4150; Fax: +44-121-685-4146

1357–714X print/1369–1643 online/01/020089-06 © 2001 Taylor & Francis Ltd
DOI: 10.1080/13577140120048593

patient following from that erroneous diagnosis. When an error had arisen then the patient or his/her next of kin, if the patient had since died, were informed. All living patients with the correct diagnosis were also informed.

### Results

A total of 1996 cases treated over the 8-year period from 1985 to 1993 at this centre were reviewed. During that time the number of new cases of suspected musculoskeletal tumours treated at the Unit rose from 108 to 341 per year—a 215% rise in work load.

The total number of errors identified was 87 cases, representing 4.4% of the total. These errors do NOT include cases where the reviewing pathologist used a different name to describe what was essentially similar pathology with no implications on that patient's management. This would include such circumstances as a fibrosarcoma being re-designated as an MFH (malignant fibrous histiocytoma) or where a Grade 2 chondrosarcoma was reclassified as a Grade 3 chondrosarcoma. Cases where the altered diagnosis may have had some clinical relevance in terms of altered management were always submitted for further review to the independent clinical advisory group. This would include such cases as identification of a dedifferentiated chondrosarcoma in a tumour previously labelled as a low-grade chondrosarcoma (the patient might have needed chemotherapy) or overdiagnosis of malignancy when the patient might have had unnecessary surgery, chemotherapy or radiotherapy.

All 87 cases were reviewed by the independent clinical advisory group, who then decided whether there had been any prejudicial effect upon that patient's treatment as a result of the misdiagnosis.

There were 21 overdiagnoses of malignancy and 36 underdiagnoses of malignancy.

In some cases the patients had the right treatment despite the wrong diagnosis, e.g., an expanding chondroid lesion was diagnosed histologically as a chondroma but on clinical grounds was treated as a malignant lesion and resected with a wide margin. Review histology confirmed that the lesion was in fact a chondrosarcoma and the patient's treatment would not have been changed.

It was concluded that, of the 87 cases where there was a difference of diagnosis, in 33 this error was of *no* clinical significance as all aspects of the treatment and follow-up were identical.

This left 54 cases where there had been a misdiagnosis which had resulted in some detriment to the patient—a clinically significant error rate of 2.7%. The incidence of these errors increased over the 8-year time span of this review (Fig. 1).

The significance of the errors was broken down into three categories depending upon the severity of the effect upon the patient. In those circumstances where there had been loss of life or limb as a result of the error in diagnosis this was labelled a 'Major' detriment. There were five Major errors.

If the patient had undergone inappropriate treatment with chemotherapy or radiotherapy or there had been a delay in diagnosis affecting the ultimate prognosis this was labelled an 'Intermediate' error. There were 17 of these.

A 'Minor' error was defined as one in which the error had caused either a delay or an adjustment to that patients treatment but which had not obviously affected the prognosis. There were 32 of these. Typical examples of this were when an initial biopsy had been reported as 'non-diagnostic' and the patient had undergone a repeat biopsy before the correct diagnosis was made. On review the original biopsy was considered to be diagnostic and hence the patient had undergone an unnecessary second biopsy. Also included are cases where the patient was underdiagnosed and *not* given chemotherapy but would normally have done so if the diagnosis had been correct. An example of this is a girl who had a lump removed from the surface of her tibia and it was diagnosed as

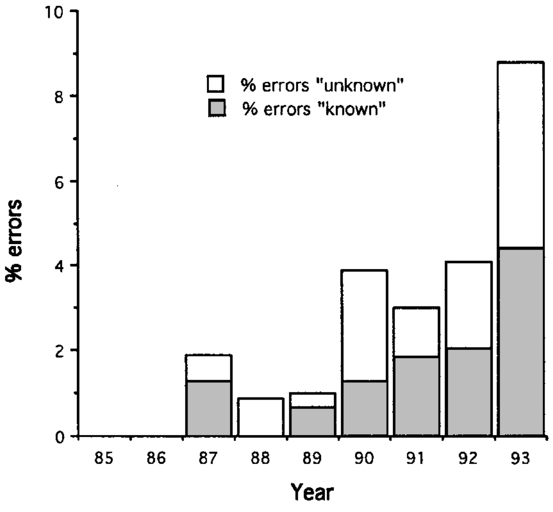

**Fig. 1.** *Graphic representation of the percentage of errors per year over the 8 years of this review. The percentage errors already 'known' are shown shaded whilst those errors revealed by this enquiry are clear.*

a chondroma. Subsequent review confirmed the diagnosis of periosteal osteosarcoma but the patient is well and without recurrence 7 years later!

Of the total 54 cases, there were two patients who may have had unnecessary amputations. Both cases were patients who had massive tumours of the pelvis and both had hemipelvectomies. In one case the tumour was diagnosed as a chondrosarcoma (Grade 3) and the amputation was done in the hope of offering a possible cure. The patient died of metastatic disease some months later. The review diagnosis was of osteosarcoma. Had this been known the patient would have been offered chemotherapy but probably not amputation because of the very poor prognosis even with chemotherapy.

Three patients had an unnecessary resection of tumour and insertion of an endoprosthesis as a result of misdiagnosis (one patient had treatment for an osteosarcoma but review showed the diagnosis to be an aneurysmal bone cyst; another had resection of a pelvic tumour thought to be MFH but on resection was found to be a plasmacytoma; whilst the third had treatment for a chondrosarcoma subsequently found to be myositis ossificans).

Seven patients had unnecessary radiotherapy following resection of a soft tissue sarcoma which on review turned out to be benign conditions (e.g., intramuscular myxomas, nodular fasciitis). Seven patients had one or more cycles of unnecessary chemotherapy as a result of the misdiagnosis (e.g., a patient with osteomyelitis diagnosed as Ewing's sarcoma). In 16 cases, patients had a second biopsy when in retrospect it was felt that the initial biopsy was in fact diagnostic (e.g., several cases where a small initial needle biopsy was felt to provide inadequate material for a firm diagnosis so an open biopsy was performed).

Some errors or misinterpretations were more common than others:

- 10 soft tissue tumours were incorrectly labelled as either benign or malignant;
- eight low-grade central osteosarcomas were all initially misdiagnosed as benign lesions;
- eight nerve sheath tumours were incorrectly interpreted as being benign/malignant;
- eight tumours were confused between osteosarcoma and aneurysmal bone cyst (both over and underdiagnosis);
- six chondroid lesions were incorrectly graded (benign/malignant/dedifferentiated);
- five eosinophilic granulomas were labelled as infection;
- five non-Hodgkin's lymphomas of bone were misdiagnosed (usually being labelled as 'reactive bone');
- three cases of osteomyelitis were misdiagnosed (as osteosarcoma or Ewing's sarcoma).

It is possible to assess the error rate for the main different diagnoses by identifying the total number of patients seen with that condition over the 8-year period and identifying the number of errors both over and under-diagnosing that condition (Table 1).

Of the 87 errors identified in this review, 25 had been known to us before the review took place. This usually occurred in cases of underdiagnosis of malignancy when the patient presented back with recurrent tumour and further biopsy confirmed the true nature of the lesion whereupon review of the original biopsy almost always confirmed that the tumour had been present all along. In other cases the resection histology was at variance with the original biopsy diagnosis and review of the biopsy again showed the presence of the correct lesion. The increasing incidence of these 'known' errors with time prompted this review (Fig. 1).

### Discussion

This review is the first histopathological peer reviewed analysis of any one musculoskeletal pathologist's work. All 1996 cases have been checked, not only by the reviewing pathologist, but also in cases of disagreement by a third independent pathologist. In all cases where there was no disagreement between the original diagnosis and the review diagnosis we believe it is reasonable to assume that an error of diagnosis is unlikely.

The difficulty of diagnosing these tumours can be emphasised by one case where a diagnosis of osteosarcoma was confidently made by a local pathologist, confirmed by a pathologist at a bone tumour registry and also by our own pathologist. The patient was reported to have small lung metastases on CT scanning. The patient was immediately started on chemotherapy and subsequently underwent resection of the primary tumour. Histology of the resected bone

**Table 1.** *Error rates for the most common tumours over the 8-year period*

| Tumour | No. treated in 8 years | Total no. of errors | No. underdiagnosed | No. overdiagnosed | Total % error rate |
|---|---|---|---|---|---|
| Osteosarcoma | 330 | 11 | 8 | 3 | 3.3 |
| Low grade osteosarcoma (includes parosteal, periosteal and low-grade central tumours) | 25 | 8 | 8 | 0 | 32 |
| Chondrosarcoma | 112 | 4 | 4 | 0 | 3.6 |
| Ewing's sarcoma | 142 | 1 | 0 | 1 | 0.7 |
| Soft tissue sarcoma | 300 | 17 | 9 | 8 | 5.6 |

showed there was no viable tumour visible and the lung lesions had completely resolved. Three years later this review changed the diagnosis to that of an aneurysmal bone cyst. This new diagnosis was confirmed by the independent review panel and a third external pathologist and it was felt that the original lung lesions were normal variants. The original radiological diagnosis of the bone lesion was consistent with either diagnosis. This case emphasises the difficulty in diagnosing some lesions and begs the question of how many opinions should be sought before commencing treatment of a presumed malignant condition in a young person.

Diagnosing bone tumours is a combination of pathology, radiology, clinical presentation and experience. It is worth noting that in many of the errors highlighted above the radiology of the lesions is non-specific and encompasses a broad differential diagnosis. Soft tissue lesions cannot be reliably diagnosed radiologically even with CT or MRI, and low-grade osteosarcomas are well known for their non-specific appearance.[9] It is not possible to grade chondroid lesions radiologically and infection is often a radiological differential diagnosis particularly for round cell tumours of bone. Hence the incidence of errors is not surprisingly highest in those very areas where radiology and clinical features are least helpful.

Mankin *et al.* have reported on errors in biopsy diagnosis twice, once in 1982 and again in 1992.[3,7] In the original study in 1982, sixteen centres with a special interest in musculoskeletal oncology reviewed 20 sequential patients who had been found to have a bone or soft tissue sarcoma. It was estimated that the biopsy was incorrect in 82/329 cases and that the error rate was 14% if the biopsy was carried out at a specialist centre, but was a staggering 40% if the biopsy was done at a referring centre. The final histopathological diagnosis was not further peer reviewed for this study. As a result of errors in diagnosis and execution of the biopsy it was estimated that 4.5% of patients had an unnecessary amputation.

In 1992 Mankin *et al.* repeated a similar study.[7] On this occasion 21 institutions supplied data on 597 malignant tumours. There was an error rate of 17.8% (106/597) of the biopsy diagnoses when these diagnoses were compared with the eventual definitive histology. Again, the final diagnosis was *not* peer reviewed. Mankin *et al.* themselves categorised these errors as major or minor depending upon their significance in terms of the patient's treatment. Eighty-one of the errors were major (13.5%), in that the error significantly affected that patient's treatment. Major errors arose in 9% of cases seen at the treatment centres and in 18.4% of patients biopsied at referring centres. In 28 cases (4.7%) the error resulted in a significant alteration in the treatment protocol, in seven of which it was believed that there had been an unnecessary amputation. There were 25 minor errors which were largely alterations of grade or nomenclature but which had little effect on management.

These two papers represent the most direct comparison with our experience, but it is interesting that in neither case was the histological material peer reviewed. It is possible to speculate therefore that the error rate would have been higher if all the material had been peer reviewed by an external assessor. Furthermore these errors are only reported for patients who were eventually found to have a diagnosis of malignancy. No mention is made of those cases which were initially *over*reported as being malignant but which subsequently turned out to be benign. In our series the rate of overdiagnosis was almost the same as that of underdiagnosis.

In their 1982 paper Mankin *et al.* have analysed their data by diagnosis and it is therefore possible to compare the error rates with those we identified (Table 2). For a better comparison we have only included in our results those cases which were under-diagnosed initially, hence these figures differ slightly to those found in Table 1.

Another method of assessing errors in diagnosis is to look at error rates in trials of treatment of bone and soft tissue tumours. Most (but unfortunately by no means all) national or international studies of bone and soft tissue tumours insist on central histopathology review as a prerequisite for entry into the study. In the European Osteosarcoma Intergroup Study the exclusion rate due to inaccurate pathology was 2.2%,[10] whilst Presant *et al.* found that 12 out of 207 (5.8%) cases entered into the South Eastern Oncology Group trials for bone or soft tissue tumours were excluded for inaccurate diagnosis.[4] The Swiss bone tumour registry documented 1100 errors in 3000 cases (36%) and reports that in 106 cases the original diagnosis of malignant was changed to benign (3.5%) and in 124 cases from benign to malignant (4.1%).[5]

Harris *et al.* reviewed 413 sarcomas diagnosed in the North West region of the UK between 1982 and 1984, and agreed with the diagnosis of sarcoma in 76% of cases but found a difference of agreement for subtyping of 47%, with the highest differences being for soft tissue sarcomas.[6] Even for bone tumours

**Table 2.** *Table to show comparative data for underdiagnosed malignant bone tumours between non-specialist and specialist centres in the USA and the present investigation: USA figures based on those in Mankin et al.*[3]

| Diagnosis | Errors at referring centre (USA) (%) | Errors at specialist centre (USA) (%) | Present series (%) |
|---|---|---|---|
| Osteosarcoma | 17 | 5 | 2.5 |
| Low-grade osteosarcoma | 60 | 50 | 32 |
| Chondrosarcoma | 42 | 14 | 3.6 |
| Ewing's | 14 | 0 | 0 |
| Overall error rate | 30.1 | 9.1 | 4.8 |

there was an error rate of 25% in patients originally diagnosed as having osteosarcoma and 20% for chondrosarcomas.

A retrospective review such as this is always likely to introduce bias. A reviewer is never under so much pressure to produce a diagnosis as the original pathologist who will be aware that a patient is actually waiting for their diagnosis and treatment will be based on the results of the biopsy. In many cases delay will already have arisen as a result of a biopsy carried out elsewhere and review of the original histological material is always essential prior to commencing treatment. This in itself causes delays and sometimes only inappropriate or inadequate specimens will get sent for review causing further delays. New techniques will be available to a reviewer (e.g., monoclonal antibodies) which simply were not accessible to the original pathologist and this too may prejudice the results of any review procedure.

All musculoskeletal pathologists accept that there will sometimes be differences between their biopsy diagnosis and the eventual diagnosis based upon resection histology.[3,7] Of major concern is the finding in this series that the actual error rate was almost twice that which the treating team had been aware of based on difference between biopsy and resection specimens. No previous study looking at error rates has ever produced data on peer-reviewed histology, and hence recognised error rates for bone and soft tissue tumour diagnosis may in fact be serious underestimates.

There are still no clear guidelines for what an 'acceptable' error rate is in histopathology, although it is generally accepted that an error rate of up to 1% in general pathology reporting is possible.[11,12] The overall error rate in this study was, in fact, the lowest ever reported for diagnosing bone and soft tissue tumours despite the rigorous nature of the review. It was the deterioration with time that prompted the review and which revealed that the actual error rate was almost twice that which was perceived.

There is little doubt that musculoskeletal histopathology is highly specialised and should not be undertaken on an occasional basis. A pathologist is just one of the team making the diagnosis and he/she should not work in isolation and should have ready access to second opinions. Participation in regular quality assurance is essential.

For all cancers the problem of underdiagnosis is one of delay in detection. The tumour will almost certainly reveal itself eventually and the diagnosis become apparent. Overdiagnosis of malignancy is the real problem which may go completely undetected and indeed be responsible for some 'miracle cures'. Entry of patients into cancer treatment trials where histopathological peer review is carried out should detect these.

This enquiry took 2 years to complete, cost over £100 000 simply for the review process and caused considerable distress and anguish to many of the 2000 patients and their relatives who had been touched by it. The costs of resolving litigation as a result of the enquiry total over £2 million.

This review has confirmed that diagnosing bone and soft tissue tumours is difficult and we firmly believe that referral to a specialist centre prior to biopsy should be the aim of all who deal with such cases.

In order to prevent instances such as this ever happening again, we would urge that all involved in managing musculoskeletal tumours should heed the lessons from this review and in particular should ensure:

1. that multidisciplinary team review of all suspected musculoskeletal tumour diagnoses is mandatory before treatment is commenced;
2. no member of the team, be it surgeon, pathologist, radiologist or oncologist should ever work in isolation;
3. that regular audit of all aspects of the Unit is mandatory, ideally involving review with other units.

**Acknowledgements**

We would like to acknowledge and thank all those involved in this review, especially the pathologists who reviewed the 1996 cases and the members of the Clinical Advisory Group.

**References**

1 Huvos AG. *Bone Tumours*. Philadelphia: WB Saunders, 1979.
2 Schajowicz F. Tumors and tumorlike lesions of bone. Berlin: Springer, 1994.
3 Mankin HJ, Lange TA, Spanier SS. The hazards of biopsy in patients with malignant primary bone and soft tissue tumours. *J Bone Joint Surg* 1982; 64A:1121–7.
4 Presant CA, Russell WO, Alexander RW, Fu YS. Soft tissue and bone sarcoma histopathology peer review: the frequency of disagreement in diagnosis and the need for second pathology opinions. *J Clin Oncol* 1986; 4:1658–61.
5 Remagen W. The importance of reference registries in bone tumour diagnosis. *Z Orthop Ihre Grenzgeb* 1992; 130:267–8.
6 Harris M, Hartley AL, Blair V, Birch JM, Banerjee SS, Freemont AJ, McClure J and McWilliam LJ. Sarcomas in North West England: histopathological Peer review. *Br J Cancer* 1991; 64:315–20.
7 Mankin HJ, Mankin CJ, Simon MA. The hazards of the biopsy, revisited. *J Bone Joint Surg (Am)* 1996; 78A:656–63.
8 Grimer RJ, Sneath RS. Diagnosing malignant bone tumours. *J Bone Joint Surg (Br)* 1990; 72B:754–6.
9 Kurt AM, Unni KK, McLeod RA, Pritchard DJ. Low grade intraosseous osteosarcoma. *Cancer* 1990; 65:1418–28.
10 Souhami RL, Craft AW, Van der Eijken JW, *et al.* Randomised trial of two regimes of chemotherpay in operable osteosarcoma: a study of the European Osteosarcoma Intergroup. *Lancet* 1997; 350:911–7.

11 Lind AC, Bewtra C, Healy JC, Sims KL. Prospective peer review in surgical pathology. *Am J Clin Pathol* 1995; 104:560–6.

12 Furness PN, Lauder I. A questionnaire based survey of errors in diagnostic histopathology throughout the United Kingdom. *J Clin Pathol* 1997; 50:457–60.

## References

[PDF_00531] Furness P. N., Lauder I. (1997). A questionnaire-based survey of errors in diagnostic histopathology throughout the United Kingdom.. J Clin Pathol.

[PDF_00518] Grimer R. J., Sneath R. S. (1990). Diagnosing malignant bone tumours.. J Bone Joint Surg Br.

[PDF_00511] Harris M., Hartley A. L., Blair V., Birch J. M., Banerjee S. S., Freemont A. J., McClure J., McWilliam L. J. (1991). Sarcomas in north west England: I. Histopathological peer review.. Br J Cancer.

[PDF_00520] Kurt A. M., Unni K. K., McLeod R. A., Pritchard D. J. (1990). Low-grade intraosseous osteosarcoma.. Cancer.

[PDF_00528] Lind A. C., Bewtra C., Healy J. C., Sims K. L. (1995). Prospective peer review in surgical pathology.. Am J Clin Pathol.

[PDF_00500] Mankin H. J., Lange T. A., Spanier S. S. (1982). The hazards of biopsy in patients with malignant primary bone and soft-tissue tumors.. J Bone Joint Surg Am.

[PDF_00515] Mankin H. J., Mankin C. J., Simon M. A. (1996). The hazards of the biopsy, revisited. Members of the Musculoskeletal Tumor Society.. J Bone Joint Surg Am.

[PDF_00503] Presant C. A., Russell W. O., Alexander R. W., Fu Y. S. (1986). Soft-tissue and bone sarcoma histopathology peer review: the frequency of disagreement in diagnosis and the need for second pathology opinions. The Southeastern Cancer Study Group experience.. J Clin Oncol.

[PDF_00508] Remagen W. (1992). Bedeutung von Referenz-Registern in der Knochentumor-Diagnostik. Erfahrungen im Schweizer Knochentumor-Register.. Z Orthop Ihre Grenzgeb.

[PDF_00523] Souhami R. L., Craft A. W., Van der Eijken J. W., Nooij M., Spooner D., Bramwell V. H., Wierzbicki R., Malcolm A. J., Kirkpatrick A., Uscinska B. M. (1997). Randomised trial of two regimens of chemotherapy in operable osteosarcoma: a study of the European Osteosarcoma Intergroup.. Lancet.

